# A review of the systematic review process and its applicability for use in evaluating evidence for health claims on probiotic foods in the European Union

**DOI:** 10.1186/s12937-015-0004-5

**Published:** 2015-02-08

**Authors:** Julie Glanville, Sarah King, Francisco Guarner, Colin Hill, Mary Ellen Sanders

**Affiliations:** 1 York Health Economics Consortium LTD, York, UK; 2 University Hospital Vall d’Hebron, Ciberehd, Barcelona Spain; 3 Alimentary Pharmabiotic Centre, University College Cork, Cork, Ireland; 4 Dairy & Food Culture Technologies, 7119 S. Glencoe Ct., Centennial, CO 80122 USA

**Keywords:** Systematic reviews, Meta-analysis, Probiotics, EFSA, Regulatory, Health claims

## Abstract

This paper addresses the use of systematic review and meta-analysis to evaluate the strength of evidence for health benefits of probiotic foods, especially relating to health claim substantiation in the European Union. A systematic review is a protocol-driven, transparent and replicable approach, widely accepted in a number of scientific fields, and used by many policy-setting organizations to evaluate the strength of evidence to answer a focused research question. Many systematic reviews have been published on the broad category of probiotics for many different outcomes. Some of these reviews have been criticized for including poor quality studies, pooling heterogeneous study results, and not considering publication bias. Well-designed and -conducted systematic reviews should address such issues. Systematic reviews of probiotics have an additional challenge – rarely addressed in published reviews - in that there must be a scientifically sound basis for combining evidence on different strains, species or genera. The European Food Safety Authority (EFSA) is increasingly adopting the systematic review methodology. It remains to be seen how health claims supported by systematic reviews are evaluated within the EFSA approval process. The EFSA Panel on Dietetic Products, Nutrition and Allergies deems randomized trials to be the best approach to generating evidence about the effects of foods on health outcomes. They also acknowledge that systematic reviews (with or without meta-analyses) are the best approach to assess the totality of the evidence. It is reasonable to use these well-established methods to assess objectively the strength of evidence for a probiotic health claim. Use of the methods to combine results on more than a single strain or defined blend of strains will require a rationale that the different probiotics are substantively similar, either in identity or in their mode of action.

## Introduction

Probiotics are live microorganisms that, when administered in adequate amounts, confer a health benefit on the host [1]. Although probiotics can be administered in different regulatory categories of products, this paper focuses on probiotics used in foods. Probiotic foods include yogurt, cheese, juices, and cereal bars among others, the most common being yogurt. European Union (EU) consumers have used probiotic foods for decades, but with the implementation of EU legislation on health claims starting in 2009, no specific health claims for probiotic foods have been approved by the agency responsible for reviewing health claim substantiation in the EU, the European Food Safety Authority (EFSA). As a result, at this point in time (September 2014), probiotic food labels cannot communicate any health benefits to consumers in the EU. Determining what level of evidence is deemed sufficient to support health claims for probiotics has been much debated in recent years [2-8]. In addition to the lack of approved health claims for probiotics in the EU, the European Commission has indicated that the term “probiotic” in itself is an implied health claim, and subsequently the term “probiotic” should not be used on products in the absence of an approved health claim [9]. Also descriptors such as “live active cultures” or “active bacteria” have been banned as descriptors for foods by some member states: Ireland [10] and Sweden [11]. Therefore, no health claims can be made on probiotic foods in the EU, even though evidence for health benefits of probiotics mounts in the scientific literature [12-16]. One reason for this seeming disparity is that studies to substantiate health claims for foods must (1) be conducted on subjects reflecting the general population, and (2) target functional or reduction of disease risk endpoints rather than therapeutic ones, [17] rendering some probiotic research ineligible to substantiate food claims. The medical community may be less inclined to make such a distinction. For example, the European Society for Primary Care Gastroenterology [18], the World Gastroenterology Organisation [19] and the European Society for Paediatric Gastroenterology, Hepatology and Nutrition [20] have all published guidelines for probiotic use.

One tool that can provide the most objective assessment of evidence on a given endpoint is a systematic review (SR), with or without a meta-analysis (MA) (the statistical combination of the results of the included studies to provide an estimate of the size of an effect or association). By systematically and rigorously identifying and critiquing as much of the available evidence for a pre-specified intervention, comparator and endpoint as possible, an assessment of the relevant body of evidence can be made. Based on this assessment, it may be possible to draw conclusions about the strength of the evidence for a specific intervention versus a specific comparator. This is a well-established approach that has been used in many fields including health and social care [21] and has also been used to explore the benefits of probiotics. Here, we review what is required for a well-conducted systematic review to set the stage for discussion of applying this method to assess evidence to substantiate a health claim for a probiotic food.

Our focus is not to build a case for any specific probiotic health claim, but to discuss the scientific basis for appropriate application of the systematic review and meta-analysis approach to assessing the totality within the field of probiotics. Although the literature is replete with meta-analyses where data on different strains and probiotic preparations have been pooled, critics hold that such techniques should be reserved for data on the same probiotic strain or strain blend. We propose that in certain well-considered situations, it is possible to pool results on different probiotics. Developing a scientific rationale based on substantial identity or common modes of action may justify such an approach. Although some probiotic functions are certainly strain-specific, we suggest conversely that not all probiotics function differently. When it is possible to link different probiotic strains by a common mechanism of action, pooling data on these strains may be appropriate.

### What are systematic review and meta-analysis?

A SR is a review that follows a pre-specified protocol to identify, select, critique, synthesize and summarize evidence to answer a focused research question [22-24]. Generally, there are seven steps to a SR [22,24-30]:

- Step 1: Framing the question
- Step 2: Identifying potentially relevant studies
- Step 3: Study selection
- Step 4: Data extraction
- Step 5: Quality assessment
- Step 6: Synthesizing the evidence
- Step 7: Interpreting the findings

In brief, the first step involves defining and refining a research question [31] followed by the development of a protocol that sets out in detail how the SR steps will be conducted. The protocol presents the research question in terms of the **P**opulation of interest, **I**ntervention(s) received by the population, **C**omparator interventions, **O**utcome(s) (or endpoints), and **S**tudy types of interest. This type of conceptual breakdown of the research question is known as ‘PICOS’. It is generally recommended that the protocol be peer-reviewed by external reviewers and then registered (e.g., through the International Prospective Register of SRs PROSPERO [32]. This is to encourage adherence to the protocol and to maximize transparency.

After the protocol is finalized, an extensive literature search is undertaken to identify relevant studies, including a search for both unpublished and published data, preferably regardless of language. Next, study titles and abstracts, and then full papers or unpublished reports are screened for inclusion in the SR with inclusion decisions made based on the defined ‘PICOS’. The data required to answer the review question are then extracted from the included studies and the methodological quality of the studies is assessed. It is recommended that study selection and data extraction are conducted by at least two reviewers working independently in order to minimize human error in making decisions about study relevance and to maximize accurate reporting of the data. To maximize consistency and reduce opportunities for error, data extraction should be piloted within spreadsheet or review software, by both reviewers, and the agreed template used for all studies. Additional data may be collected from the original researchers who conducted the included studies [33].

Quality assessment of the included studies is a key component of the SR process as it provides a view on the reliability of the evidence reported in each of the studies. There are a number of quality assessment and risk of bias tools available [22,24,34-38] and they are usually specific to the study type (i.e., a different quality assessment tool asking different questions would be used for randomized trials than for non-randomized observational studies).

Depending on the nature of the outcome data, the results of SRs may be presented in a narrative and/or quantitative synthesis. When it makes sense to combine data from similar studies (i.e., when they have similar participants, interventions and outcomes) it may be possible to quantitatively synthesize the outcomes using statistical techniques such as meta-analysis (MA). MA pools results from different studies to obtain an average estimate of effect across studies. In simple terms, studies may be weighted so that larger studies have a greater impact on the pooled result than smaller studies. This method increases the statistical power to detect a difference in effect that may not be detected in individual studies, and increases the precision of the estimate of the effect of the intervention [22,24]. Inevitably, there will be some variation in the estimated effects between studies included in a MA. If the variation is significant (statistically heterogeneous) subgroup analyses may be conducted. Ideally these should be specified *a priori* in the case of study characteristics that would be expected to influence treatment effects. Subgroup analyses are also used to explore inconsistencies between study results that are unlikely to have arisen by chance alone [22]. Sensitivity analyses may also be conducted (for example, omitting studies with lower quality from the analysis) to give an indication of the ‘robustness’ of the results [39]. Meta-regression is an extension to subgroup analysis that evaluates continuous and categorical variables [24].

The last step of the SR process includes interpretation of the findings in the context of the quality of the body of evidence [24,27]. If the SR includes a MA, the degree of consistency across studies should be considered to increase confidence in the pooled estimate of effect [35]. Without knowing the consistency of the results between studies (i.e., the degree of heterogeneity), it is impossible to determine the generalizability of the estimate for the average effects [35]. At this stage, the main results are also discussed within the context of other (systematic) review evidence and any gaps in the evidence.

When reporting a SR, transparent reporting of the review methods and assumptions should be the main objective. SRs should conform to reporting guidance such as the Preferred Reporting Items for Systematic Reviews and Meta-Analyses (PRISMA) framework [28]. Ideally, systematic reviews should also be subject to peer review.

### Use of systematic review and meta-analysis methods in assessing the effects of probiotics

The SR process is used in several arenas to assess the evidence base in support of regulatory and policy recommendations. Despite the robustness of SR methodology for assessing a body of evidence, we identify four issues relating to the use of meta-analysis in the field of probiotics:

- Was the intervention defined appropriately?
- Was the search extensive and was publication bias assessed? SRs which have not searched widely for studies and which have not assessed publication bias may produce biased results.
- Were results combined appropriately?
- Were analyses conducted to assess study quality? SRs and MAs that do not explore the effect of including lower quality studies run the risk of providing unreliable results.

#### Was the intervention defined appropriately?

Defining the intervention can be a particular challenge for SRs of probiotics, which comprise a broad range of different genera, species and strains of live microbes. An intervention, or the class of substance (such as a probiotic) that is the subject of the review, does not have to be a single substance. But in the case of grouping multiple substances into one class, there must be a sound rationale that the members of the class as defined are expected to function in a similar manner (Table 1). A probiotic intervention may comprise a single strain or a mixture of strains selected from a broad range of live microorganisms, including prokaryotic and eukaryotic microbes of different genera, species and strains. Common bacterial genera used as probiotics include *Lactobacillus*, *Bifidobacterium*, *Propionibacterium*, *Streptococcus* and *Bacillus*. Strains of the yeast, *Saccharomyces cerevisiae* (biovariant *boulardii*), are also used.

**Table 1:** Criteria that may be acceptable* for combining different probiotic strains into the same ‘class of intervention’ for a specific outcome

Rationale	Probiotic formulation defining the intervention class	Example	Comment
Common identity	Single strain of one specific genus and specie	*L. acidophilus* strain A	All studies include the same strain; may include studies conducted in different food matrices
Common identity	Single defined blend of multiple strains (strains A + B + C)	*L. acidophilus* strain A and *L. casei* strain B and *B. lactis* strain C	Strains must be maintained in equivalent relative doses in all studies
Common taxonomy	Studies used different strains of same species or subspecies?	*L. acidophilus* strain A, *L. acidophilus* strain B, or *L. acidophilus* strain C	Different studies using different strains included
Common structural or secreted product (e.g., beta-glycosidases, exopolysaccharides)	Different strains from a defined taxonomic group that may include different species or genera	All strains of *L. bulgaricus* and *S. thermophilus*	Different studies using different strains included
Common mechanism of action known to be necessary and sufficient for the effect (e.g., production of a specific bacteriocin or range of bacteriocins known to be active against a specific pathogen, or induction of immune mechanisms needed for the effect)	Different strains from a defined taxonomic group that may include different species or genera	*L. salivarius* strain A	Different studies using different strains included
		*L. salivarius* strain B	
Common physiological effect previously proven by at least one human study of quality	Different strains from a defined taxonomic group that may include different species or genera	All strains	Different studies using different strains included

*To the extent that commonalities cannot be defined for different probiotic strains, studies on them should not be pooled into a meta-analysis. For example, probiotics such as *Saccharomyces boulardii*, *Bacillus coagulans* and *Bifidobacterium* species likely are too different biologically and mechanistically from each other to form one class of intervention. Additionally, even if taxonomically similar, if different strains have different mechanisms responsible for an effect, it may be inappropriate to pool studies. For example, a meta-analysis on anti-infective actions of probiotics likely should not pool data from a *Lactobacillus rhamnosus* strain where effects are known to be due exclusively through immune enhancing activity in the small intestine and a *Lactobacillus salivarius* strain where effects are known to be due primarily through bacteriocin production in the colon.

Observations by early researchers on probiotic functionality, especially with regard to outcomes from *in vitro* or animal studies, clearly indicate that different strains of the same species may behave differently. Van Hemert et al. [40] illustrate this point. They tested 42 *Lactobacillus plantarum* strains and found a 14-fold difference in the strains’ ability to induce peripheral blood mononuclear cells to secrete interleukin-10. Similar strain-specific responses are seen in many other *in vitro* and animal studies that assess biological properties of different probiotics. The body of literature in human studies reveals few head-to-head comparisons of probiotics. A few examples are available in the literature where different probiotic products or different genera or species are compared. Canani et al. [41] compared 5 different probiotic preparations with a placebo and found that two, but not the other three, were effective in treating children with acute diarrhoea. O’Mahony et al. [42] compared a *Lactobacillus salivarius* strain and a *Bifidobacterium infantis* strain in subjects with irritable bowel syndrome and found that only the *B. infantis* strain significantly alleviated symptoms. Such studies show that in some cases, different clinical outcomes result from using different strains. However, in other conditions, different strains of probiotics have a similar impact on a particular clinical endpoint. This is the case with necrotizing enterocolitis [43], antibiotic-associated diarrhoea [14] and upper respiratory tract infections [12], where SRs combining results of multiple probiotic preparations resulted in convincing evidence of efficacy for a broad range of probiotics. In these examples, not all tested probiotics seemed equivalently effective, but different probiotics were effective. In fact, many SRs published on probiotics have tended to combine all probiotic preparations, although criticism of this approach is building [19]. Some SRs that have included a wide range of probiotic strains conclude that not all strains are equally effective [44]. A few SRs have focused on single strains, such as *L. rhamnosus* GG [45,46], *S. boulardii* [47] or *L. reuteri* [48].

A scientific justification may exist for grouping multiple strains, species or even genera of probiotics into one class of intervention for the purpose of conducting a SR and MA. It may be appropriate to group multiple strains of a narrow taxonomic cluster, such as *Bifidobacterium animalis* subsp. *lactis* where evidence shows little diversity in the members of this subspecies. Lee and O’Sullivan state that four strains of *B. animalis* subsp. *lactis* exhibited >99% sequence identity over their entire genomes, indicating a very closely related group [49]. Masco et al. [50] have also shown that based on pulsed-field gel electrophoresis, all commercial *B. lactis* strains are indistinguishable. Barrangou et al. [51] also found a high degree of genome conservation indicative of a monomorphic subspecies. Collado et al. [52] showed that 7 of 8 *B. lactis* strains had essentially the same strain-specific pattern as indicated by a random amplified polymorphic DNA technique. Such taxonomic grouping may, however, be criticized in the absence of information on the mechanism of action for the benefit, since it is possible that the part of the genome that is not shared encodes the functional activity.

It may also be appropriate to propose that several probiotics might be legitimately grouped based on the production of common “structures”, such as peptidoglycan, flagellae or exopolysaccharides, known to evoke specific physiological responses in the host or could be grouped based on a common mechanism of action. This latter example is the case with the yogurt cultures, where aiding lactose digestion is linked to the microbial production of beta-galactosidase. EFSA approved the health claim that “yogurt cultures” (which encompass any strains from the species *Lactobacillus bulgaricus* and *Streptococcus thermophilus*) could improve lactose digestion [53]. Note that this claim does not extend to the *Lactobacillus* genus, as mixed results have been reported among other tested *Lactobacillus* species. In another example, it is conceivable that a class could be defined as members of a specific taxonomic cluster that also produce certain levels of a certain metabolite *in vivo*, such as specific short chain fatty acids.

When clustering different probiotics into one intervention group, the conclusions of the SR could only apply to the intervention class as defined. Therefore, if the intervention was defined as the subspecies, *B. animalis* subsp. *lactis*, rather than *B. animalis* subsp. *lactis* strain Bb-12, for example, then the conclusions of the SR would apply to all strains of *B. animalis* subsp. *lactis*, whether tested in a human study or not. This concept has already been accepted by EFSA in the case of yogurt cultures. The presumption that any benefits apply only to the individual strains used in the included studies reflects a lack of conviction by those defining the class as the subject of the SR that the class is logically and appropriately defined. Where strains express unique characteristics (not common to their genera or species) that enable a specific health benefit, they should not be grouped in a MA since results from one strain cannot inform (or predict) effects of another strain of the same genus or species. However, if there is a scientifically sound basis for defining a class that encompasses different strains, information on all members of that class can be used to provide a more comprehensive set of supporting data. If a study does not show an effect for one of the strains in the class, this may simply represent a study with null results, some of which will be expected in any body of research. If a strain is not represented in the collection of reviewed studies, it also does not mean that it should be excluded from the general conclusions on the benefit or otherwise of that class. Alternatively, subgroup analysis that shows efficacy for specific members of the defined class may enable researchers to refine the class more narrowly. Of note to this discussion is that in theory, EFSA accepts pooling data from studies of a well-defined food constituent that is responsible for a claimed effect when given at the appropriate dose [54]. However, if pooling data on different probiotic products (comprising different strains), we reiterate that it will be necessary to provide a scientific justification for considering this group as a defined class.

Challenges emerge if one intends to use pooled data from studies on different strains clustered into one intervention group as part of a dossier to substantiate an EU health claim. One pillar of such a dossier is characterization of the substance under consideration. This section clearly defines what food or food ingredient is the subject of the health claim. EFSA requires that characterization is very specific. A strategy for definition of the substance under review must be developed if data from different strains, potentially delivered in different matrices, are to be combined in a MA. It is possible that a SR for a defined set of probiotics can be used as evidence for a dossier for one specific member of that class. Then the characterization information should be specific for that one member.

#### Was the search extensive and was publication bias assessed?

Although the SR might be useful to bring the studies together, the MA should not be undertaken when there is likely to be serious publication bias, since this is likely to provide an unreliable estimate of the true effect size [24]. Publication bias occurs when publication is influenced by the results. An extensive search for both published and unpublished data and the use of a range of different search techniques mitigates this issue, but relevant studies may still remain inaccessible to specific reviews. This is why investigations of publication bias, where possible, are helpful in revealing the possible extent of missed studies and the potential for risk of publication bias should be considered within the conclusions of a SR [55]. Prospective trial registries such as ClinicalTrials.gov improve awareness of research that has been undertaken and already includes numerous probiotic trials.

#### Were studies combined appropriately?

When deciding whether to conduct a MA, input from a topic expert is often helpful to ascertain whether it is appropriate to combine the results of the identified studies [24]. Current guidance recommends that data should not be pooled when there is a mix of different comparisons of different treatments, or when the outcomes are too diverse (i.e., when they measure different variables) [24]. In the case of probiotics, this guidance would not preclude combining studies on different probiotic products as long as there is a solid scientific rationale for grouping the different probiotics into one class. As an extension to this, it may be misleading to combine results in a MA if there are substantial differences between study estimates of effect, particularly if they are in opposing directions [22]. If this occurs with probiotics studies, the studies could be grouped into logical subsets, for example by probiotic strain, species, or genera (defined *a priori)*.

#### Were analyses conducted to assess study quality?

MAs of poor quality studies may result in an erroneous interpretation [24]. Different methods have been suggested to deal with this potential problem, such as only including high quality studies in a review, or conducting sub-group analyses, or sensitivity analysis by quality criteria, to allow a focus on the higher quality studies. Quality assessment may reflect concerns about study methods, but may also take into account other aspects of the study such as concerns about bias caused by the funding of the research or inadequate information about the study as a result of the publication format (in the case of conference abstracts). The inclusion of *only* poor quality studies in MA (or where a high proportion of the weight of the evidence is from poor quality studies) should be avoided, as they may cast doubt on any subsequent conclusions, or even hinder any correct interpretation.

### Use of the systematic review/meta-analysis process by the European Food Safety Authority

In 2010, EFSA published its guidance on the conduct of SRs of relevance to food and feed safety [23]. EFSA noted that, although rarely used in these areas, SR methods had potential for application in the fields of food and feed safety. In 2012, EFSA advertised a series of framework agreements (Scientific Services to Support EFSA Systematic Reviews) [56] with suppliers to provide training in SR methods, to offer expert support at various stages of the SR process and to provide SRs to EFSA. Suppliers were requested to perform SRs in line with the methods described in the EFSA framework agreements [56].

The EFSA guidance [23] clearly stresses the importance of precisely defined, closed-framed questions (exposure or intervention, diagnostic accuracy, descriptive questions of populations or systems), which are captured *a priori* in clear eligibility criteria for studies to be included in the review.

In cases where there is a large quantity of evidence, the SR method can formally and systematically summarize that evidence (with MA where data permit) and provide more precise estimates of effects than an individual study can provide. The value of the SR approach also lies in its presentation of all of the available evidence with an assessment of the quality of that evidence: the strengths and limitations of the evidence base can thus be seen in a clearer way. The more controversial the topic, the more important it is for the SR process to be described in detail, so that the methods can be understood and alternative approaches discussed. The EFSA guidance provides a clear outline of a suitable process for conducting a SR and some recommendations on the reporting of a SR, without providing a formal reporting structure such as the PRISMA guidance described earlier. In the recent framework tender documents [56], suppliers are asked to consider documenting and reporting the method and results of the SR using the PRISMA statement and, where that is not applicable, to document and explain any discrepancies from the PRISMA statement.

The use of MA within SRs is guided by the suitability of the data for pooling; the considerations informing a decision to use MA are described in Appendix E of the EFSA guidance [23]. Although this is not a formal recommendation of the EFSA guidance, the presentation of the issues around MA in the guidance suggests that for SRs to be as transparent as possible, the rationale and justification for MA should be presented in the SR report. The discussion and conclusions of the SR should be grounded in the *a priori* objectives for the SR and the results identified. Again, adhering to this approach and documenting the rationale in full will contribute to the rigour and objectivity of a particular SR.

### Use of the systematic review and meta-analysis for evaluation of evidence to substantiate health claims on foods in the European Union

In Europe, Article 6 of EC Regulation 1924/2006 states that health claims for food labeling must be based on and substantiated by ‘generally accepted scientific evidence’ [57]. In order to ensure that health claims made are truthful and can be understood by consumers, health claims undergo a specific procedure of assessment and authorization involving consultation to the EFSA Panel on Dietetic Products, Nutrition and Allergies (NDA Panel) [57]. For the assessment of all types of health claim applications (article 13.1, 13.5 or 14), the NDA Panel considers whether the beneficial effect of the food is substantiated by generally accepted scientific evidence [57] using an assessment process of the highest possible standard. As described by the NDA Panel, this involves taking into account the totality of the available scientific data, and weighing the evidence obtained from individual studies [57].

The EFSA ‘General guidance for Article 13.1, 13.5 and 14 health claims evaluation’ [57] indicates that data from intervention and observational studies in humans should be presented with the most reliable data being assessed according to a hierarchy of study designs. Trials with full randomization and adequate allocation concealment (method of randomization reported) are deemed to be at the top of the hierarchy of human intervention studies for assessing a cause-effect relationship between the consumption of the food or food constituent and the claimed health effect [58]. Cohort studies are considered to be at the top of the hierarchy for human observational studies [58].

While the NDA panel did not propose a formula for how many or what type of studies are needed to substantiate a claim, the reproducibility of the effect of the food as indicated by consistency between studies is an important consideration for their final assessment. Thus, a comprehensive review of the data from human studies addressing the specific relationship between the food and the claimed effect is required from the applicant.

The NDA panel guidelines make it clear that in evaluating and weighing the evidence to substantiate health claims on foods, SR and MA can play an important role [58]. EFSA ask applicants to provide a comprehensive review in order to evaluate the totality of the evidence proposed in the dossier. This step must be included as a specific section of a health claim dossier. The NDA panel’s final opinion will be based on a SR of the totality of the available data, but does not require that all individual randomized trials or other type of studies show statistical significance. For example, when considering the evidence for periconceptional folate supplementation for preventing birth defects, 5 randomized trials were considered [59]. Since the incidence of birth defects was quite low, the MA process allowed pooling of available data to reach a convincing conclusion about the effectiveness of folate. The MA reached a level of evidence that was not reached by the individual randomized trials.

The NDA panel does require that the SR provides convincing evidence on the consistency and reproducibility of the effect when analyzing the totality of the data. EFSA considers randomized trials as the best tools to generate evidence on the effects of interventions and considers SR and MA to be the best tools to evaluate the totality of the evidence and inform a final opinion. MA and SR may be useful to address the following scenarios that we envision could emerge in submitted dossiers:

- Studies are underpowered due to a smaller effect size than predicted, so none of the studies reaches statistical significance. But, when data are analyzed together as part of a MA, a statistically significant average result is obtained.
- One pivotal study has been conducted which is in favor of the intervention, and other studies with null results have been published. This reflects a genuine evidence base and the review of all contributing studies should allow exploration of heterogeneity and provide information on the true direction and size of the health benefit. All studies, with all results, contribute to an overall picture of the effect.
- Studies are underpowered due a heterogeneous population within each study, comprising responders and non-responders, which cancel each other out in the analysis of the individual study. The best approach to this situation is to better identify the target population *a priori* and perform randomized trials with the appropriate population subset, or to unpick the studies into the relevant subgroups. On the other hand, acceptance of the health claim has the potential to benefit a subset of the population consuming the product, which is of benefit to those consumers. Subgroup analyses defined *a priori* may enable identification of the responding subgroup and convincing evidence in support of a claim for the subgroup may be revealed.
- The incidence of the health endpoint is low (such as incidence of flu during the winter season) and very large sample sizes among the study population are required. Such sample sizes are better achieved by pooling the results of several randomized trials, providing that these studies collectively provide sufficient power.
- Many statistically significantly positive randomized trials exist, but no two trials investigate the same strain. If there is a scientifically valid basis to expect that a group of different strains would function similarly on an endpoint, then they can be reasonably grouped into one intervention for a meta-analysis, and the resulting meta-analysis would be sufficient evidence to support a health claim.

We recognize that SRs are only one input to a decision-making process regarding formulation of policy derived from evidence. The health technology assessment process, for example, also considers information on the economic and legal aspects of competing interventions.

## Conclusions

SR and MA are transparent and rigorous approaches to synthesizing evidence. It is widely accepted in many scientific disciplines and by many policy-setting organizations as the best way to evaluate the strength of evidence available to answer a focused question. EFSA guidance acknowledges the validity of well-conducted SRs for closed-framed questions, which seems appropriate for assessing evidence for probiotics: *In population P, does intervention I affect outcome O when assessed using comparator C?*

EFSA endorses the SR process to inform their judgment when deciding health claims, as observed with the decision on maternal folate intake and reduced risk of neural tube defects. This suggests the method is also suitable for probiotic health claims.

Applying the SR approach to probiotics to obtain evidence on a single strain (e.g., Strain A) or on a single, defined multi-strain probiotic (e.g., Strain A + B + C) is scientifically justifiable. Applying the SR approach to studies conducted on a broader range of probiotics (i.e., studies of different strains of the same species or subspecies, different species or different genera, such that study 1 is conducted on strain A, study 2 is conducted on strain B, study 3 is conducted on strain C + D) requires a scientifically valid justification for definition of the class. However, EFSA precedent has allowed such ‘clustering’ of all yogurt cultures into a category of food that can improve lactose digestion, even though the possibility exists that a particular strain may not deliver adequate lactase to improve lactose digestion. EFSA seems to recognize that available evidence provides a reasonable certainty that yogurt cultures will provide this benefit. However, 100% certainty is not possible and should not be required.

The majority of SRs published on probiotics to date has been conducted on a broad group of live microorganisms without stated justification for clustering into one class. In many cases, this includes studies using *Lactobacillus*, *Bifidobacterium*, *E. coli*, *Saccharomyces* and others. If the overall effect is positive, the conclusion from such reviews has generally been that ‘probiotics’ are effective for the particular indication. However, they have not concluded that every possible probiotic strain will necessarily be effective, and they have generally acknowledged that effects may be due to only specific, tested strains. A strict application of the SR approach should enable application of the conclusions of the review to every member of the class as defined.

Procedures within EFSA, including their approach to definition and characterization of the substance under review, presents a challenge for use of the SR and MA approaches on a defined class of different strains of probiotics for primary health claim substantiation.

In conclusion, SR and MA represent a well-developed and widely applied means to evaluate the strength of research evidence and should be acceptable by EFSA for substantiating probiotic claims. Use of SR to combine studies on multiple probiotic strains, however, requires a valid scientific rationale that combining evidence from different strains is biologically and physiologically justified.

## Abbreviations

EFSA: European food safety authority
EU: European Union
MA: Meta-analysis
NDA Panel: Panel on Dietetic Products, Nutrition and Allergies
PRISMA: Preferred reporting items for systematic reviews and meta-analyses
SR: Systematic review
